# Supplementary material for: Stacking Model‐Based Classifiers for Dealing With Multiple Sets of Noisy Labels
Source: Biom J. 2025 Mar 12;67(2):e70042. doi: 10.1002/bimj.70042 (PMC11898607; doi:10.1002/bimj.70042)
Supplement: Supplementary file 1 — Supporting Information [file BIMJ-67-e70042-s001.zip › density-based-ensemble-model-main/competitors_real_data/netlab-master/netlab-master/help/somfwd.htm]

Netlab Reference Manual somfwd


# somfwd

## Purpose

Forward propagation through a Self-Organising Map.

## Synopsis

```
d2 = somfwd(net, x)
```

## Description

`d2 = somfwd(net, x)` propagates the data matrix `x` through
a SOM `net`, returning the squared distance matrix `d2` with
dimension `nin` by `num_nodes`. The $i$th row represents the
squared Euclidean distance to each of the nodes of the SOM.

`[d2, win_nodes] = somfwd(net, x)` also returns the indices of the
winning nodes for each pattern.

## Example

The following code fragment creates a SOM with a $5times 5$ map for an
8-dimensional data space. It then applies the test data to the map.

```
net = som(8, [5, 5]);
[d2, wn] = somfwd(net, test_data);
```

## See Also

`som`, `somtrain`

---

**Pages:**
Index


---

Copyright (c) Ian T Nabney (1996-9)
